# MicroRNA-340-5p inhibits colon cancer cell migration via targeting of RhoA

**DOI:** 10.1038/s41598-020-73792-9

**Published:** 2020-10-09

**Authors:** Anwar Algaber, Amr Al-Haidari, Raed Madhi, Milladur Rahman, Ingvar Syk, Henrik Thorlacius

**Affiliations:** Section for Surgery, Department of Clinical Sciences, Skåne University Hospital, Lund University, 20502 Malmö, Sweden

**Keywords:** Cancer, Tumour immunology

## Abstract

Colon cancer is the third most common cancer and a significant cause of cancer-related deaths worldwide. Metastasis is the most insidious aspect of cancer progression. Convincing data suggest that microRNAs (miRs) play a key function in colon cancer biology. We examined the role of miR-340-5p in regulating RhoA expression as well as cell migration and invasion in colon cancer cells. Levels of miR-340-5p and RhoA mRNA varied inversely in serum-free and serum-grown HT-29 and AZ-97 colon cancer cells. It was found transfection with miR-340-5p not only decreased expression of RhoA mRNA and protein levels in HT-29 cells but also reduced colon cancer cell migration and invasion. Bioinformatics analysis predicted one putative binding sites at the 3′-UTR of RhoA mRNA. Targeting this binding site with a specific blocker reversed mimic miR-340-5p-induced inhibition of RhoA activation and colon cancer cell migration and invasion. These novel results suggest that miR-340-5p is an important regulator of colon cancer cell motility via targeting of RhoA and further experiments are warranted to evaluate the role of miR-340-5p in colon cancer metastasis.

## Introduction

Colon cancer is one of the most common types of tumors and the third leading cause of death among cancer patients in Europe^1^. Metastatic spread of tumor cells is the most dominant cause of mortality in colon cancer patients^2^. The underlying mechanisms of metastasis remain unclear, however, a strong body of evidence suggests that increased expression of adhesion molecules and chemokines facilitate colon cancer cell migration and spread^3^. The migratory machinery of cancer cells is governed by complex interactions between specific cell cytoskeletal proteins coordinated by small GTPases, such as the Rho family of proteins (RhoA-C, Cdc42 and Rac-1)^4^. RhoA plays an important function as a molecular switch in transducing extracellular signals to actin and microtubule cytoskeleton as an integrated part of cell migration^5^. Interestingly, activation of Rho proteins is a very commonly observed prooncogenic signal and overexpression of RhoA has been reported to be associated worse prognosis of patients with colorectal cancer^6^. Thus, knowledge about mechanisms regulating RhoA could be useful to develop strategies to antagonize colon cancer cell migration and invasion.

MicroRNAs (miRNAs) are short-run of non-coding RNAs (21–23 nucleotides) and function as posttranscriptional regulators of gene expression^7^. An increasing number of studies have demonstrated aberrant miRNAs expression in cancers and that miRNAs regulate important function in cancer progression, including cell adhesion, migration, invasion, proliferation and apoptosis by targeting specific oncogenes or tumor suppressor genes^8^. Accumulating data have shown that miR-340-5p is down-regulated in glioblastoma, prostate, breast, gastric, osteosarcoma and colorectal cancer cells^9–14^, suggesting an important role of miR-340-5p in cancer progression. In fact, studies have revealed that miR-340-5p inhibits growth^14–16^ and increases apoptosis^14,15^ and chemosensitivity^14^ of colon cancer cells. In a study by Takeyama et al. (2014)**,** it was found that low expression of miR-340-5p correlated with shorter 5-year disease-free survival and poor 5-year overall survival. In this context, it is interesting to note that miR-340-5p has been found to target RhoA in squamous and non-small cell lung carcinoma as well as melanocytes^17–19^ although the interaction between miR340 and RhoA in colon cancer cells remains elusive.

Based on the considerations above, we hypothesized that miR-340-5p might regulate colon cancer cell migration and invasion via targeting of RhoA. Therefore, we used human colon cancer cell line transfected with miR-340-5p mimic to evaluate its functional role on motility.

## Methods and materials

### Cells and reagents

HT-29, human epithelial colon adenocarcinoma cell line was obtained from American Type Culture Collection (HTB-38, ATCC, Manassas, VA, USA). We have established a primary human colon cancer cell line in our laboratory at Skåne University Hospital called AZ-97, which was isolated from a 76-year-old female patient undergoing surgical resection as previously described^20^ and these cells have been shown to have metastatic capacity^21,22^. Cells were cultured in Dulbecco’s Modified Eagle Medium (DMEM)(Sigma-Aldrich, Stockholm, Sweden), supplemented with 10% fetal bovine serum (FBS) and antibiotics (100 U/ml penicillin, 100 μg/ml streptomycin) at 37 °C and 5% CO2. Mimic-miR-340-5p and mimic-ctrl (Life Technologies, Carlsbad, CA, USA) were used to evaluate the role of miR340-5p by use of *TransIT-TKO* transfection reagent (Mirus; Madison, WI, USA). Target site blocker (TSB) LNA oligonucleotides were purchased from Exiqon A/S (Vedbaek, Denmark).

### Cell transfection

Cell transfection was done as described earlier^23^. HT-29 colon cancer cells at 70–80% confluency were serum starved overnight and then 1 × 10^6^ cells were seeded in a 6-well culture plate. Cells were then transfected with mimic-miR-340-5p (25 nM or 50 nM) or mimic-Ctrl (50 nM) for 24 h or 48 h by using Mirus transfection reagent in Opti-MEM reduced serum media according to the manufacturer’s instructions. 24 h after transfection, cells were harvested and expression of miR-340-5p and RhoA mRNA was analyzed by RT-qPCR. Briefly, Cells were lysed by using TRIzol (Invitrogen; Thermo Fisher Scientific, Inc.) and then RNA samples were extracted using Direct-zol RNA MiniPrep extraction kit (Zymo Research, Irvine, CA, USA) according to the manufacturer’s recommendations. 0.4 µg of total RNA was used to synthesized cDNA in each reactions using Mir-X miRNA First-Strand Synthesis Kit for miR-340-5p and RevertAid First Strand cDNA Synthesis kit for RhoA. miR-340-5p and RhoA mRNA were quantified using TB Green Advantage qPCR Premix (Clontech, Mountain View, CA, USA). The PCR primers used were as follows; hsa-miR-340-5p specific sense 5′-GGCTTATAAACGAATCACAGTCATTAAAA-3′, RhoA mRNA sense; 5′-AGAGGTGTATGTGCCCACAGTGTT-3′, antisense; 5′-AGGCGATCATAATCTTCCTGCCCA-3′, U6 sense; 5′-GCTTCGGCAGCACATATACTA-3′, U6 antisense; 5 CGAATTTGCGTGTCATCCTTG-3′, Beta actin sense; 5′-AGAG CCTCGCCTTTGCCGATCC-3′, antisense; 5′-CACATG CCGGAGCCGTTGTCG-3′. 2^-ΔΔCT^ method was used to determine expression of RhoA mRNA and miR-340-5p relative to beta actin and U6 snRNA.

### Target site prediction and target site blockers (TSB) of miR-340-5p

Bioinformatics analysis using the Target Scan prediction tool predicted only one binding site for miR-340-5p at the 3′-UTR of RhoA mRNA which is conserved between different species, (https://www.targetscan. org/). To evaluate the function of the binding site, we designed target site blockers, TSB (20 nucleotides) to bind selectively to the 8′mer seeding sequence of miR-340-5p in the 3′-UTR of RhoA mRNA. To increase the affinity and selectivity of the TSB it was synthesized as fully phosphorothiolated Locked Nucleic Acids (LNA) in the DNA sequences. Under serum starved conditions, the target site blocker TSB_RhoA_miR-340-5p; 5′-TTATAAAGTAGTTACAGCCT-3′ were co-transfected with the mimic-miR340-5p in different concentrations (12.5–50 nM). The levels of RhoA mRNA was later quantified using qRT-PCR as described above.

### Chemotaxis assay

Colon cancer cell migration was evaluated using 24-well cell migration chambers with 8 μm pore size inserts (Corning Coster, Corning, NY, USA). Colon cancer cells were serum**-**starved overnight and transfected with either mimic-miR-340-5p (50 nM), mimic-Ctrl (50 nM), TSB, and TSB-Ctrl for 24 h in Opti-MEM serum reduced media as previously described^24^. Briefly, cells were transfected with mimic-mir340-5p, mimic-Ctrl, TSB (50 nM), and TSB-Ctrl (50 nM) for 24 h. After transfection, 1 × 10^6^ cells/ml were loaded in the inserts and DMEM with 10% serum in the lower chambers and incubated for 24 h at 37 °C (5% CO_2_). Non-migrated cells were removed from the upper surface of the insert and cells on the lower surface of the insert membrane were fixed in ice-cold 100% methanol. After washing with PBS, cells were stained with 0.5% crystal violet. In separate experiments, cells were pre-incubated for 30 min with the Rho kinase inhibitor, Y-27632 (50 µM) (R&D systems Europe, Abingdon, UK). Cells were counted microscopically by using high power field (HPF) in five different fields. Data were expressed as the mean number of migrated cells per high power field.

### Invasion assay

Cell invasion was determined by using 24-well cell chambers with 8 μm pore size inserts (Corning Coster, Corning, NY, USA) coated with 30 μg of ECM Gel (Sigma-Aldrich, MO, USA) per well. Colon cancer cells were serum-starved overnight and transfected with either mimic-miR-340-5p (50 nM), mimic-Ctrl (50 nM), TSB, and TSB-Ctrl for 24 h in Opti-MEM serum reduced media as described above. After transfection, 1 × 10^6^ cells/ml were seeded in the upper well of the invasion chamber in serum-free condition. The lower chamber well contained DMEM supplemented with 10% FBS to stimulate cell invasion. Non-invading cells were removed from the top well with a cotton swab, while the bottom cells were fixed with 100% methanol, stained with 0.1% crystal violet. In separate experiments, cells were pre-incubated with the Rho kinase inhibitor, Y-27632 (50 µM) (R&D systems Europe, Abingdon, UK) for 30 min. The cells counted microscopically by using high power field (HPF) in five different fields. Data are expressed as the mean number of invaded cells per high power field.

### Total and active RhoA assay

Cells were serum**-**starved overnight and transfected by either mimic-miR-340-5p (50 nM) or mimic-ctrl (50 nM) for 24 h. The activity of RhoA-GTP was measured using the G-LISA RhoA Activation Assay Biochem kit (Cytoskeleton Inc., Denver, CO, USA) according to manufacturer's instructions as previously described^23^. Total RhoA was measured using RhoA ELISA Biochem Kit (Cytoskeleton Inc., Denver, CO, USA) according to manufacturer's instructions. Briefly, cells were grown to 60% confluence and then serum**-**starved overnight. Next day cells were stimulated by 10% FBS for 30 min at 37 °C. Cells were washed twice by ice cold PBS and lysed according to manufacturer’s protocol for 10 min on ice. After lysis, cells were homogenized using a 20-gauge needle on ice and then centrifuged at 14,000 g for 5 min at 4 °C. Total protein concentration was determined using Precision Red Advanced Protein Assay supplied with the kit. 1 mg/ml of protein was used to for quantitative detection of active and total RhoA according to the manufacturer’s instructions. Luminescence signal was detected using a microplate luminescence reader.

### Statistical analysis

GraphPad Prism 8 was used for statistical analysis and data were presented as mean values ± standard error of the mean (SEM). For multiple comparisons, Kruskal–Wallis One Way Analysis of variance of ranks followed by the Dunnett’s post hoc test was used and for two groups comparison, the Mann Whitney test was used. *P*-value < 0.05 was considered significant.

## Results

### MiR-340-5p negatively regulates RhoA mRNA expression in colon cancer cells

**Figure 1:**
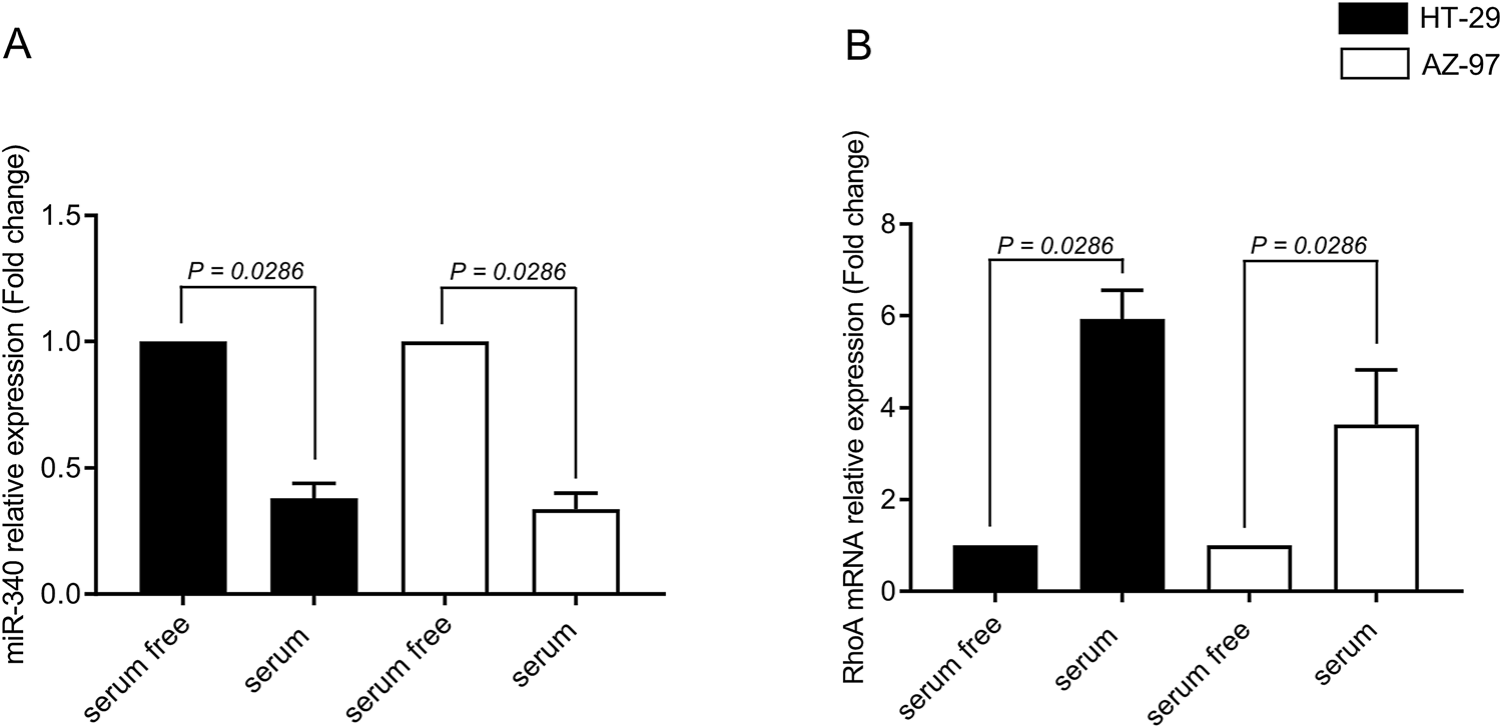
Gene expression of miR340-5p and RhoA in HT-29 and AZ-97 colon cancer cells lines. Expression of (**A**) miR-340-5p and (**B**) RhoA mRNAs and the housekeeping gene U6 were determined using qRT-PCR in serum-free and serum-grown HT-29 cells. Relative expressions were demonstrated using qRT-PCR where U6 was used as a housekeeping gene for mir-340-5p and beta-actin was used as a housekeeping gene for RhoA mRNA and expressions were determined using 2–ΔΔCT method. Data represent mean ± SEM and *n* = 4.

**Figure 2:**
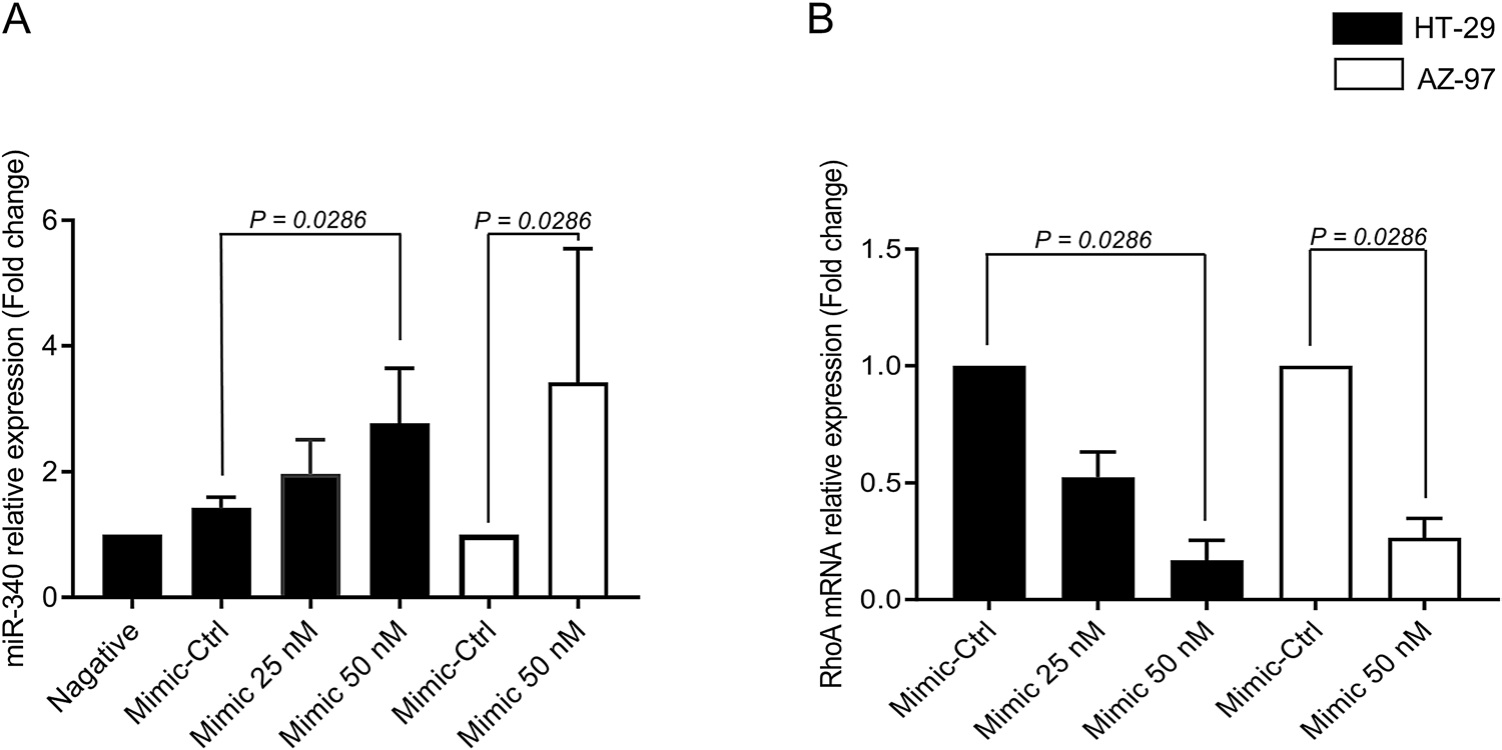
Mir-340-5p regulates RhoA mRNA expression in HT-29 and AZ-97 colon cancer cells. Transfection with Mimic-Ctrl (50 nM) or miR-40-5p mimic (25 nM and 50 nM) (**A**) upregulates miR-340-5p and (**B**) downregulates RhoA mRNA expression in colon cancer cells. Relative expressions were demonstrated using qRT-PCR where U6 was used as a housekeeping gene for mir-340-5p and beta-actin was used as a housekeeping gene for RhoA mRNA and expressions were determined using 2^–ΔΔCT^ method. Data represents mean ± SEM and (*n* = 4).

Serum-free conditions have been shown to mimic stress conditions of the tumor microenvironment^25–27^. Expression of miR-340-5p in HT-29 and AZ-97 colon cancer cell lines were assessed in both serum-free (to mimic stress condition of tumor microenvironment) and serum-grown culture conditions using RT-qPCR. It was found that levels of miR-340-5p was significantly lower in serum-grown compared to serum-free cultured cells (Fig. 1A). Moreover, RhoA mRNA expression was found to be higher in serum-grown compared to serum-free (Fig. 1B), suggesting a negative correlation between miR-340-5p and RhoA mRNA in colon cancer cells. Serum-grown tumor cells were used for the following experiments in this study. We next transfected cells with a mimic control and a mimic 340-5p and found that transfection with the mimic 340-5p for 24 h increased levels of miR-340-5p (Fig. 2A). Concomitantly, transfection with mimic 340-5p dose-dependently decreased Rho mRNA levels in colon cancer cells (Fig. 2B). Similar results were observed when HT-29 cells were transfected with miR-340-5p mimic for 48 h (Supplementary Figure 1A,B).

### RhoA is a direct target of miR-340-5p

**Figure 3:**
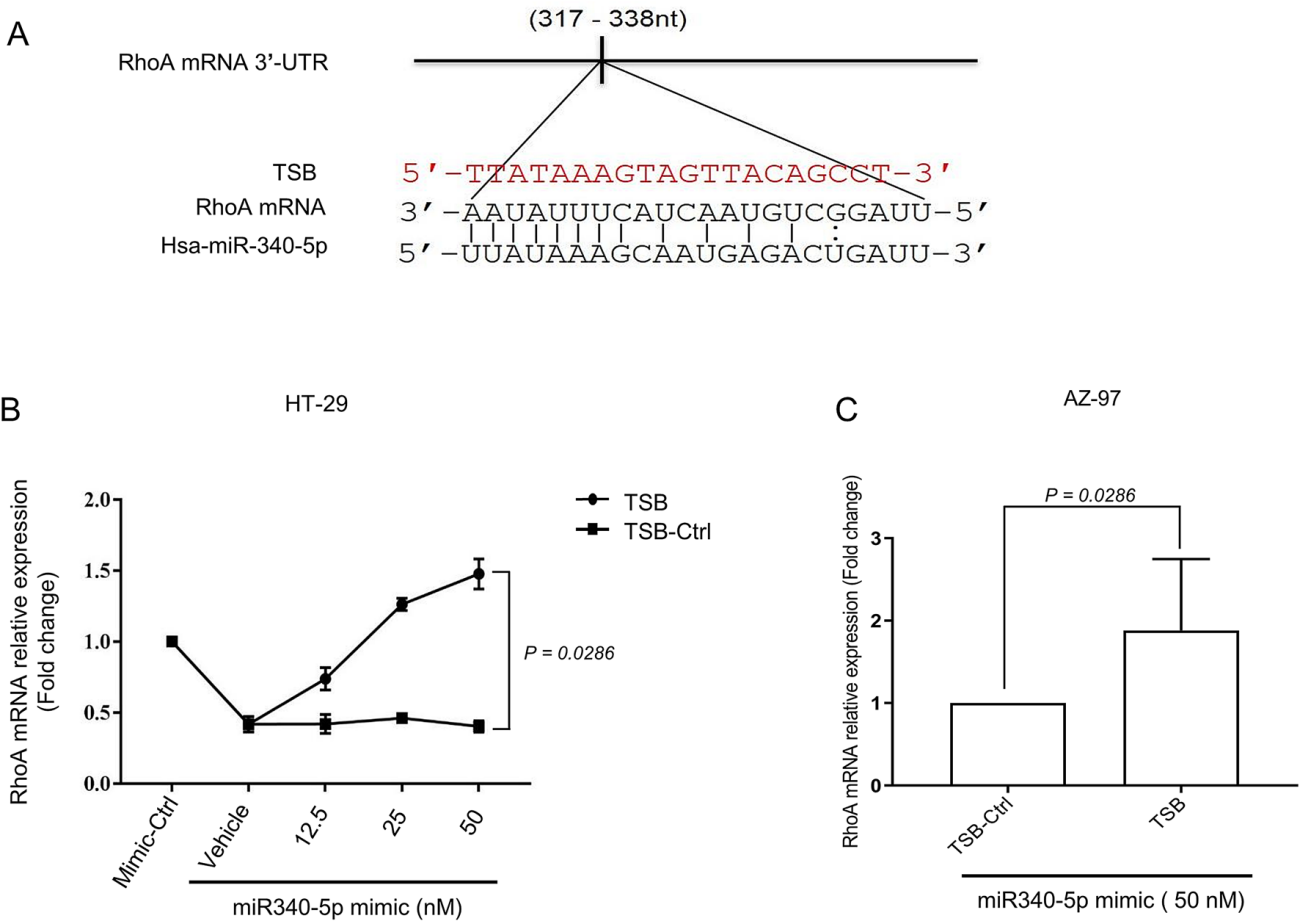
RhoA is a direct target of miR-340-5p. (**A**) Predicted target site of miR-340-5p in RhoA mRNA 3′-UTR sequence containing an (AAUAUUUC) motif. The seeding region of miR-340-5p complementary to (UUAUAAAG) was blocked using TSB (red sequence). (**B**) TSB dose-dependently reversed the inhibitory effect of miR-340-5p on RhoA mRNA expression in HT-29 colon cancer cells. (**C**) TSB dose-dependently reversed the inhibitory effect of miR-340-5p on RhoA mRNA expression in AZ-97 colon cancer cells. Data represent mean ± SEM and (*n* = 4).

Next, we asked whether RhoA could be a target of miR-340-5p in colon cancer cells. Bioinformatics analysis using the public database target site prediction tool (TargetScan) revealed that miR-340-5p has one potential binding site at RhoA mRNA 3′-UTR where it contains complementary sequences of perfect 8′mer base-pair match to the seeding region of miR-340-5p (Fig. 3A). Binding of miR-340-5p to this target site on RhoA mRNA was validated by a specific target site blocker (TSB). Again, RhoA expression was markedly decreased in HT-29 cells after transfection with miR-340-5p mimic (Fig. 3B). Notably, co-transfection of HT-29 cells with TSB reversed the inhibitory effect of miR-340-5p mimic on RhoA mRNA in a dose-dependent manner. In contrast, co-transection with a control TSB had no effect on the expression of RhoA mRNA in colon cancer cells transfected with miR-340-5p mimic (Fig. 3B). Similar results were observed when AZ-97 cells were co-transfected with TSB and miR-340-5p mimic (Fig. 3C).

### MiR-340-5p inhibits colon cancer cell migration and invasion by targeting RhoA activity

**Figure 4:**
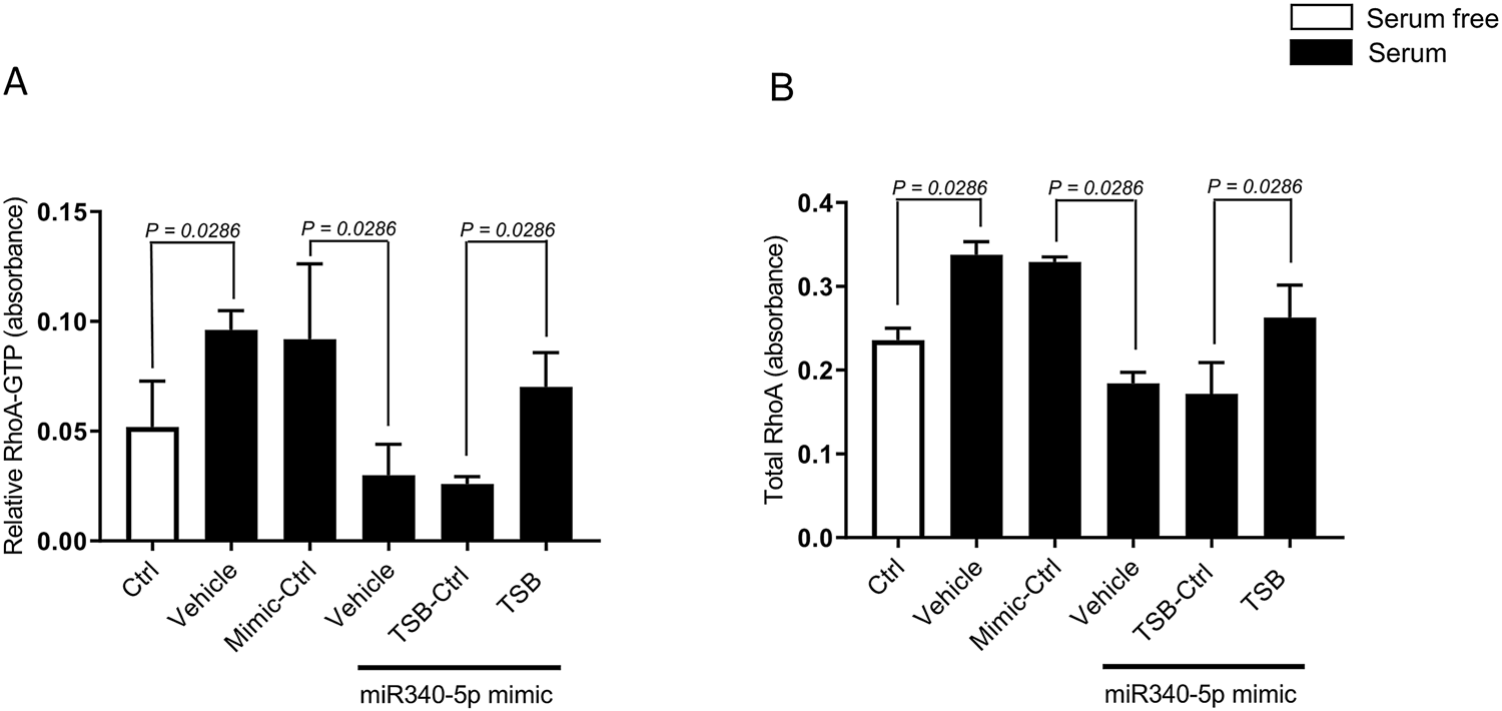
Mir-340-5p regulates colon cancer cell migration and RhoA activation. (**A**) RhoA-GTP activation was quantified using the G-LISA activation assay kit. (**B**) Total RhoA was measured by total RhoA assay kit. Cells were transfected with miR-340-5p mimic, mimic control, TSB control and TSB. Data represent mean ± SEM and *n* = 4.

**Figure 5:**
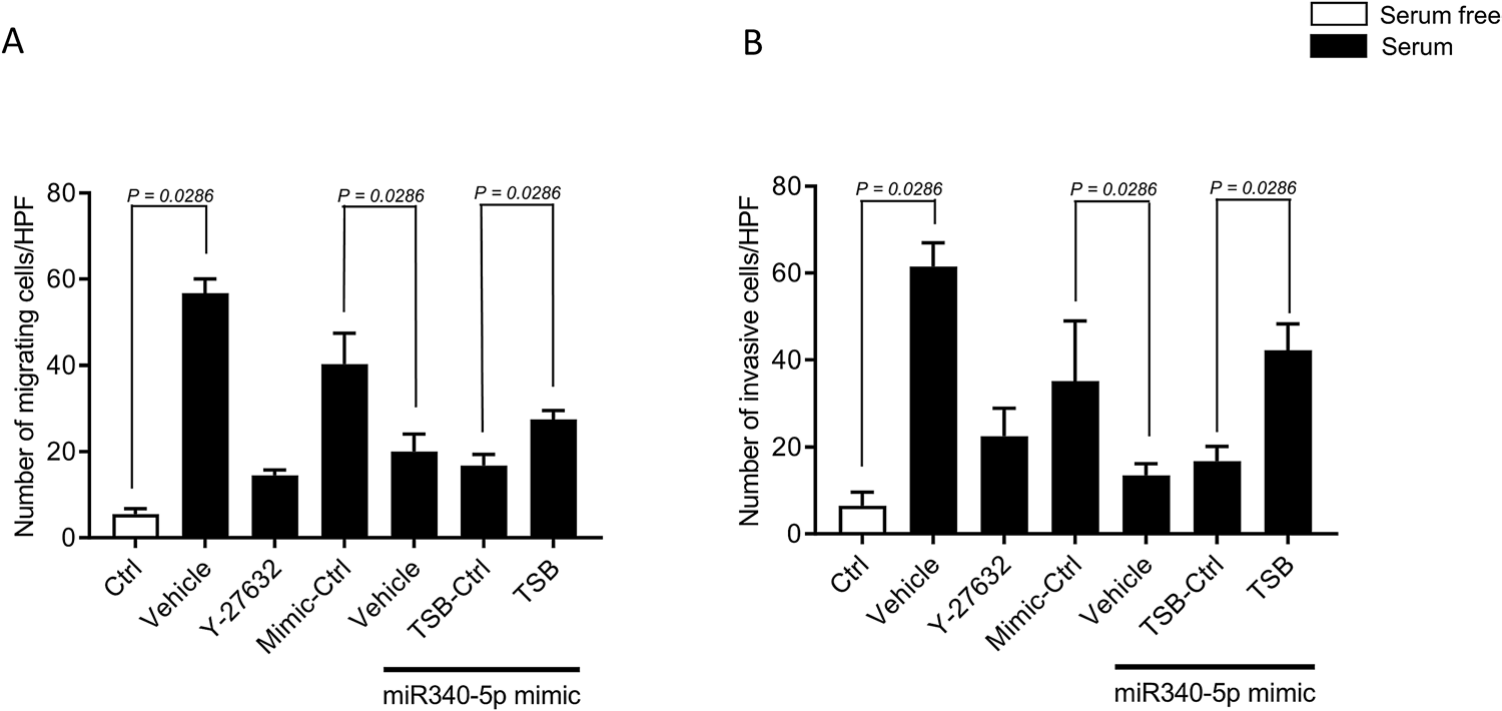
(**A**) Migration of colon cancer cells were stimulated by use of 10% FBS. Cells were counted microscopically using high power fields in five different fields. (**B**) Invasion of colon cancer cells were stimulated by use of 10% FBS. Cells were transfected with miR-340-5p mimic, mimic control, TSB control and TSB. In one group, cells were pre-incubated with the Rho kinase inhibitor Y-27632 (50 µM) for 30 min before loading into the inserts. Cells were counted microscopically using high power fields in five different fields. Data represent mean ± SEM and *n* = 4.

Transfection of HT-29 cells with miR-340-5p mimic decreased both total RhoA and Rho-GTP activity completely (Fig. 4A,B). Notably, it was found that co-transfection with the TSB, but not the TSB control, reversed miR-340-5p mimic-induced inhibition of and total RhoA and Rho-GTP activity in colon cancer cells (Fig. 4A). To examine whether miR-340-5p regulates colon cancer cell migration and invasion by targeting RhoA, transwell migration and invasion assays were performed using 10% serum as a chemoattractant. It was found that serum addition significantly increased colon cancer cell migration and invasion (Fig. 5A,B; Supplementary Figures 2, 3). Administration of the Rho-kinase inhibitor Y-27632 significantly decreased colon cancer cell migration and invasion (Fig. 5A,B). Transfection with miR-340-5p mimic significantly reduced colon cancer cell migration and invasion (Fig. 5A,B; Supplementary Figures 2, 3. Moreover, we observed that co-transfection with the TSB, but not the TSB control, increased migration and invasion in colon cancer cells transfected with miR-340-5p mimic (Fig. 5A,B; Supplementary Figures 2, 3). Transfection of HT-29 cells with miR-340-5p mimic and control mimic had no effect on cell numbers compare to non-transfected control (Supplementary Figure 4).

## Discussion

Tumor cell migration and invasion are prerequisites for subsequent metastasis to distant organs^28^. This study demonstrates that miR-340-5p negatively regulates colon cancer cell migration and invasion by targeting RhoA. By use of a specific target blocker we identified the specific binding site of miR-340-5p on RhoA mRNA. Thus, our findings suggest that miR-340-5p could be used to antagonize spread of colon cancer cells to distant organs.

Accumulating data suggest that miRNAs constitute a critical class of short noncoding RNAs regulating an array of cellular functions, such as differentiation, growth, angiogenesis proliferation, adhesion, migration, invasion and apoptosis in cancer progression^29,30^. Multiple specific miRs have been shown to regulate certain aspects of the metastatic process of colon cancer^31^. The role of microRNA in metastatic colorectal cancer and its significance in cancer prognosis and treatment. For example, Takeyama et al. (2014) reported that expression of miR340 was lower in colon cancer tissue compared normal adjacent tissue. Interestingly, they observed that decreased expression of miR-340-5p correlated with increased incidence of liver metastasis^32^, indicating a role of miR340 in the metastatic process of colon cancer cells. In the present study, we could demonstrate that transfection of colon cancer cells with miR-340-5p mimic reduced the chemotactic response, suggesting that miR-340-5p has the capacity to negatively regulate colon cancer cell migration. Moreover, we observed that overexpression of miR-340-5p decreased colon cancer cell invasion. These findings could help to explain the increased numbers of liver metastasis in patients with low levels of miR-340-5p. Our results showing that miR-340-5p controls colon cancer cell migration is in line with previous studies showing that migration of breast, lung and squamous cancer cells is attenuated by miR-340-5p^17,18,33^. Moreover, our data suggest that miR-340-5p also regulates colon cancer cell invasion. In this context, it also important to note that colon cancer cell growth^16^, apoptosis^15^ and chemosensitivity^14^ is dependent on miR-340-5p. Together with our present findings these studies suggest that miR-340-5p regulates colon cancer progression at multiple levels.

MicroRNA control gene expression via targeting of specific sites on transcribed mRNAs^34^ . Previous work has documented that miR-340-5p targets REV3L^15^, c-Met^32^ and RLIP76^14^ in colon cancer cells, leading to growth inhibition. It is widely held that RhoA function as a potent proto-oncogene and is frequently overexpressed in various kinds of tumors, including colon cancer^35^. RhoA exerts a key role as a molecular switch in transducing extracellular signals to actin and microtubule cytoskeleton, constituting an integrated part of the cell migration machinery^5^. Knowing that RhoA plays a key role in regulating cancer cell migration^23,35,36^, it was of great interest, herein, to investigate the potential role of miR-340-5p in regulating RhoA in colon cancer cells. Notably, it was observed that Y-27632 markedly decreased colon cancer cell migration and invasion, indicating that Rho-kinase is involved in the motility of colon cancer cells. Next, we found that transfection with miR-340-5p mimic significantly reduced RhoA mRNA and protein levels as well as RhoA-GTP activity in colon cancer cells, suggesting that RhoA is a target of miR-340-5p in colon cancer cells. This observation is supported by three previous studies showing that miR-340-5p can target RhoA in squamous carcinoma cells^17^, non-small cell lung cancer^18^ and melanocytes^19^. In order to clarify if RhoA is a direct target of miR-340-5p, a bioinformatics analysis was performed. One potential binding site on RhoA mRNA which complementary to a sequence region of miR-340-5p was identified. We then designed a specific blocker targeting the identified binding region of the 3´-UTR of RhoA mRNA. Interestingly, it was found that co-incubation with this specific blocker dose-dependently reversed mimic miR-340-5p-induced inhibition of RhoA mRNA expression, indicating that this specific region of 3´-UTR of RhoA mRNA is a functional target of miR-340-5p in colon cancer cells. Thus, this study identifies a target site regulating translational inhibition of RhoA mRNA by miR-340-5p in CRC. In addition, this specific TSB also reversed mimic miR340-induced inhibition of colon cancer cell migration and invasion, suggesting that this specific site on RhoA mRNA is functional target of miR-340-5p with a potential significance for the metastatic process of colon cancer. It should be mentioned that RhoA is an upstream regulator of Rho kinase (ROCK1 and ROCK2), which has been shown to control colon cancer cell migration^35^ and that two previous studies have shown that miR-340-5p targets ROCK1 in osteosarcoma and breast cancer cells^13,37^. Whether miR-340-5p also can inhibit ROCK1 remain to be demonstrated. Finally, it is important to state that our findings do not exclude that miR-340-5p might target other molecules, such as RhoC and Cdc42, of potential importance for colon cancer cell migration and invasion.

In conclusion, this study demonstrates that miR-340-5p negatively regulates colon cancer cell RhoA activity as well as migration and invasion. Moreover, this effect of miR-340-5p was found to mediate by specific elements present in the 3´-UTR region of RhoA mRNA. Our results not only show how miR-340-5p affects colon cancer cell motility but might also serve to help developing new and effective strategies against colon cancer cell metastasis.

### Supplementary information

Supplementary Figures.

## Abbreviations

miRNAs: MicroRNAs
TSB: Target site blocker
FBS: Fetal bovine serum
HPF: High power fields
LNA: Locked nucleic acids
DMEM: Dulbecco's modified eagle medium
ROCK: Rho-associated protein kinase
