# Author Correction: MicroRNA-340-5p inhibits colon cancer cell migration via targeting of RhoA

**DOI:** 10.1038/s41598-024-53419-z

**Published:** 2024-02-15

**Authors:** Anwar Algaber, Amr Al‑Haidari, Raed Madhi, Milladur Rahman, Ingvar Syk, Henrik Thorlacius

**Affiliations:** Section for Surgery, Department of Clinical Sciences, Skåne University Hospital, Lund University, 20502 Malmö, Sweden

Correction to: *Scientific Reports* 10.1038/s41598-020-73792-9, published online 09 October 2020

The Supplementary Figures file published with the original version of this Article contains errors.

As a result of an error during figure assembly, panel Y-27632 in Supplementary Figure 2 and panel Y-27632 in Supplementary Figure 3 are incorrect.

The correct Supplementary Figures file is now linked to this correction notice.

### Supplementary Information

Supplementary Figures.

