# Supplementary material for: Author Correction: MicroRNA-340-5p inhibits colon cancer cell migration via targeting of RhoA
Source: Sci Rep. 2024 Feb 15;14:3849. doi: 10.1038/s41598-024-53419-z (PMC10869820; doi:10.1038/s41598-024-53419-z)
Supplement: Supplementary file 1 — Supplementary Figures. [file 41598_2024_53419_MOESM1_ESM.pdf]

MicroRNA-340-5p inhibits colon cancer cell migration via targeting of RhoA

Anwar Algaber, Amr Al-Haidari, Raed Madhi, Milladur Rahman, Ingvar Syk and Henrik Thorlacius

48 hours transfection

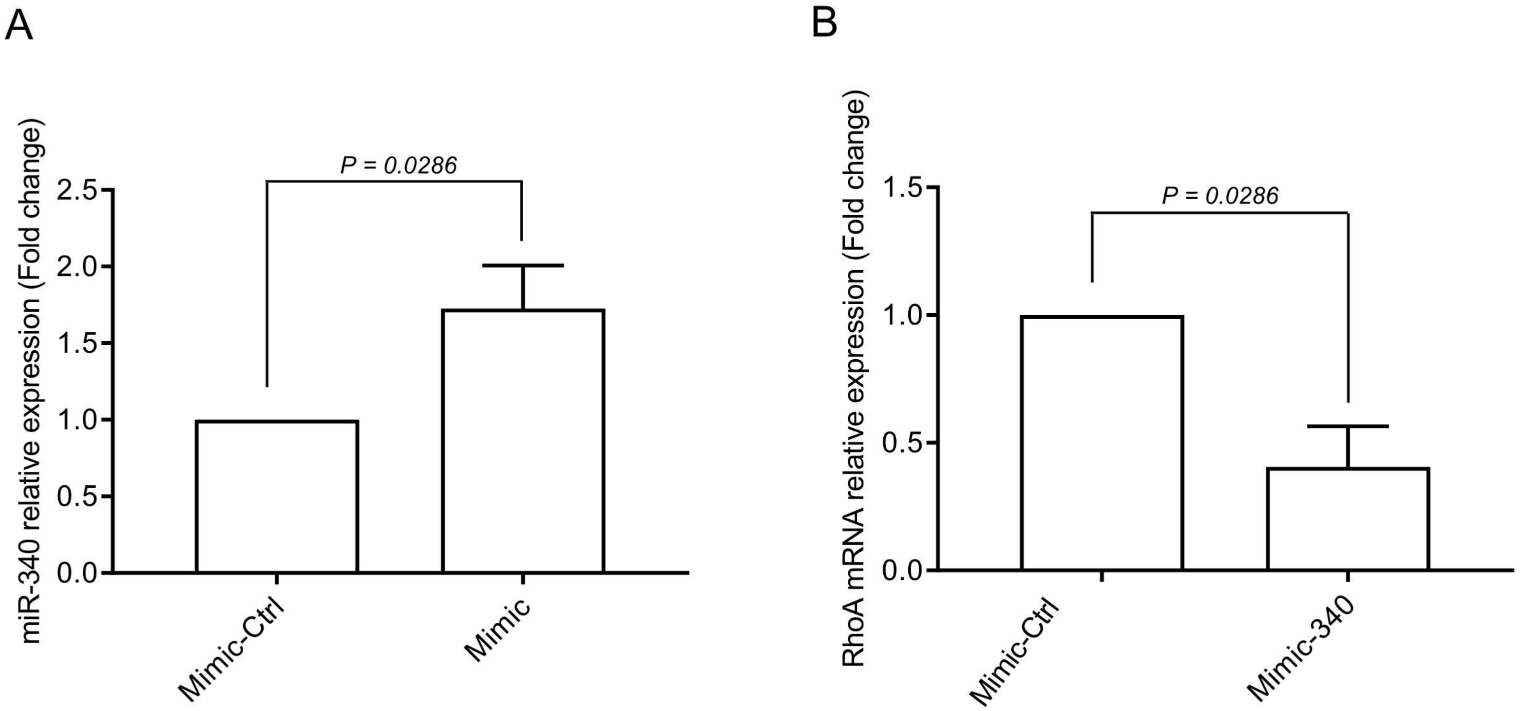

Supplementary Figure 1. Mir-340-5p regulates RhoA mRNA expression in colon cancer cells. Transfection with Mimic-Ctrl (50 nM) or miR-40-5p mimic (50 nM) for 48 h. A) upregulates miR-340-5p and B) downregulates RhoA mRNA expression in HT-29 cells. Relative expressions were demonstrated using qRT-PCR where U6 was used as a housekeeping gene for mir-340-5p and beta-actin was used as a housekeeping gene for RhoA mRNA and expressions were determined using $2^{-\Delta\Delta CT}$ method. Data represents mean ± SEM and (n = 4).

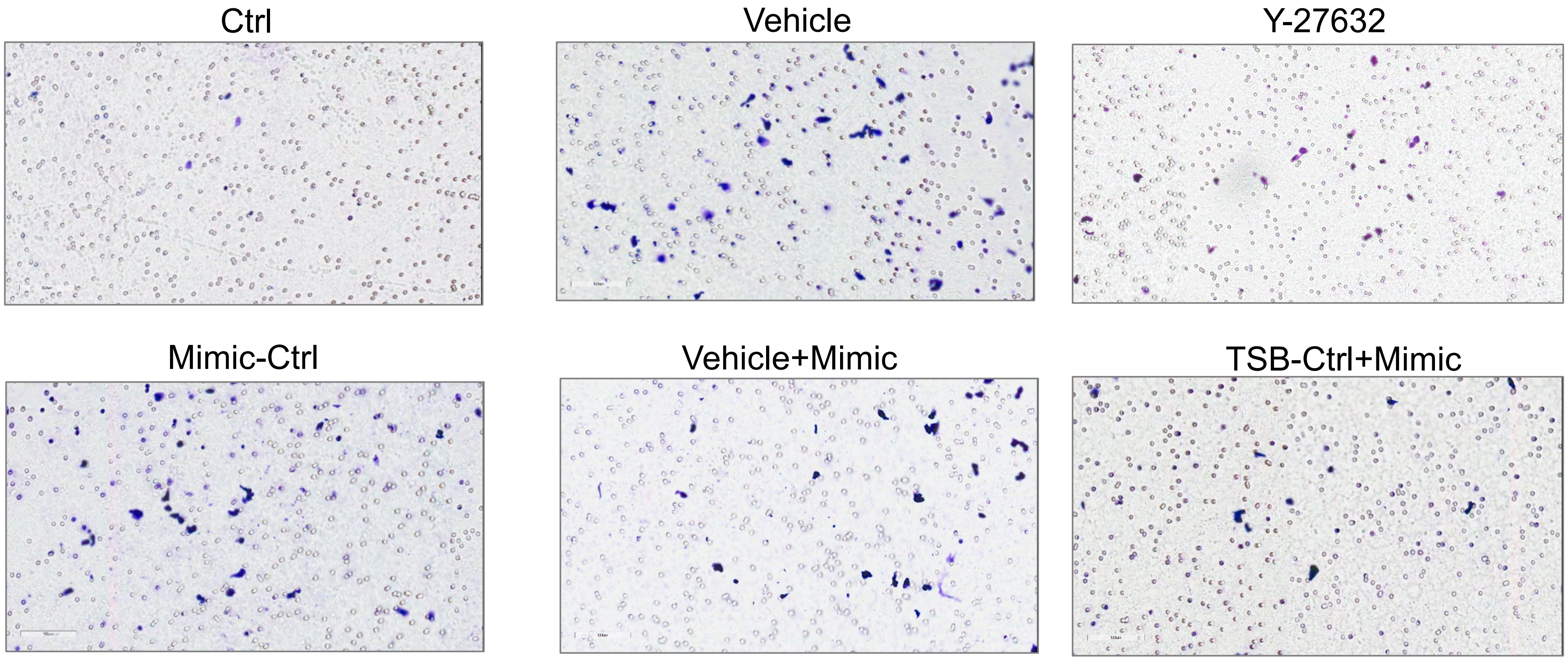

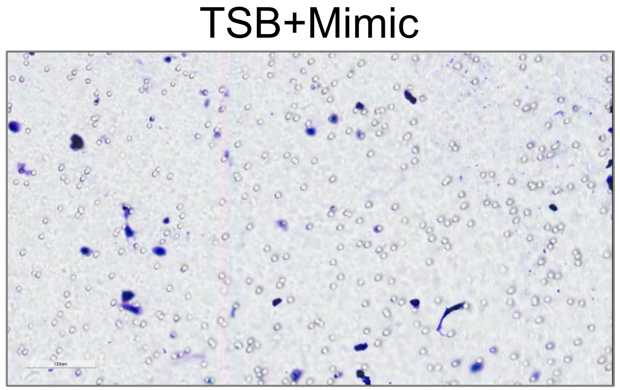

Supplementary Figure 2. Representative images of migration assay of Figure 5A. Migration of colon cancer cells (HT-29) were stimulated by use of 10% FBS. Cells were transfected with miR-340-5p mimic, mimic control, TSB control and TSB. In one group, cells were pre-incubated with the Rho kinase inhibitor Y-27632 (50 μM) for 30 min before transfection. Cells were counted microscopically using high power fields in five different fields.

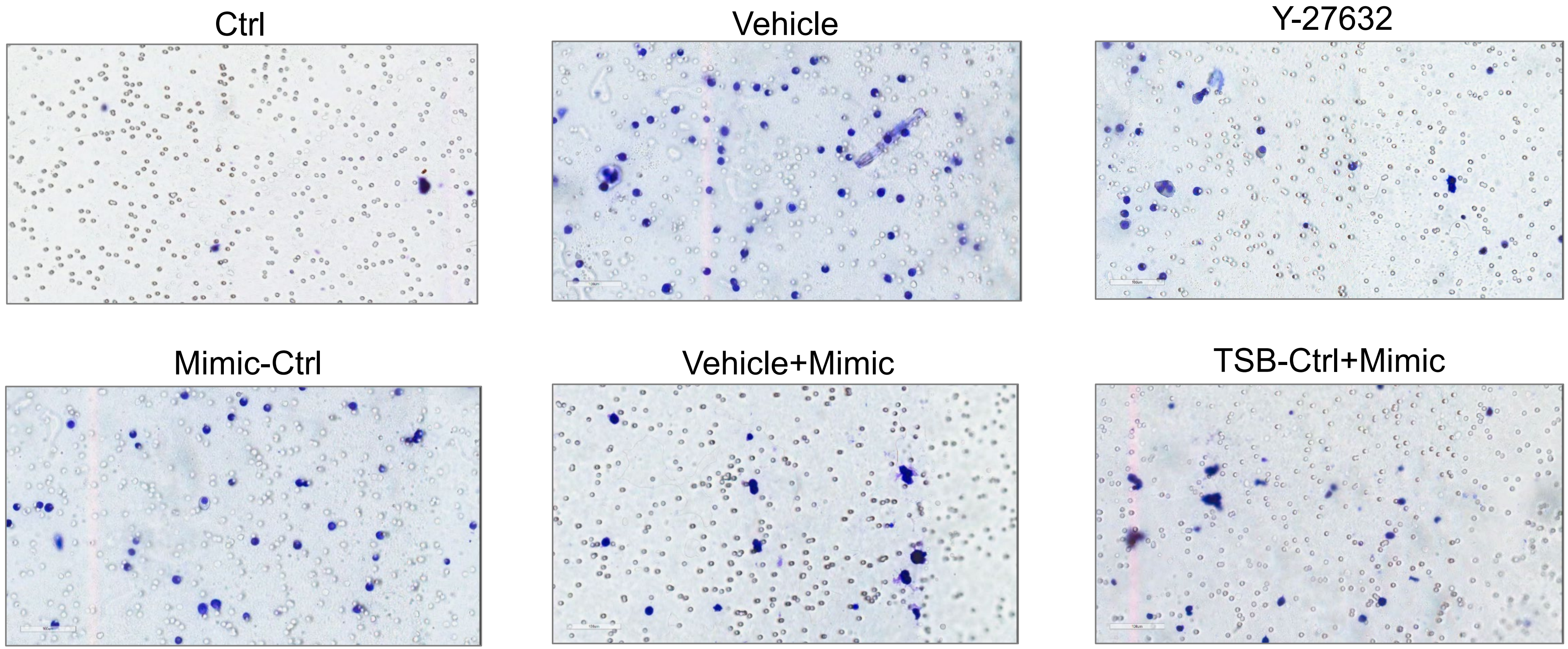

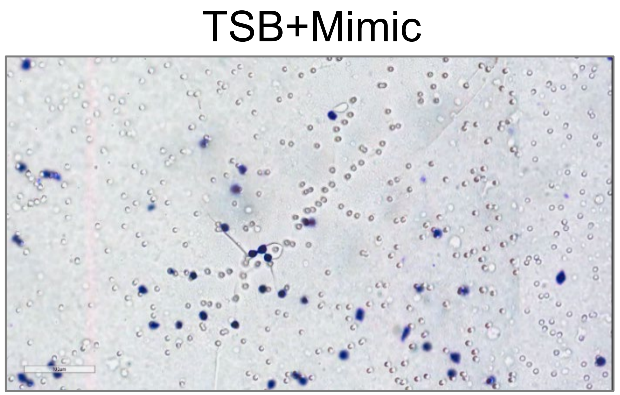

Supplementary Figure 3. Representative images of invasion assay of Figure 5B. Invasion of colon cancer cells (HT-29) were stimulated by use of 10% FBS. Cells were transfected with miR-340-5p mimic, mimic control, TSB control and TSB**.** In one group, cells were pre-incubated with the Rho kinase inhibitor Y-27632 (50 μM) for 30 min before transfection. Cells were counted microscopically using high power fields in five different fields.

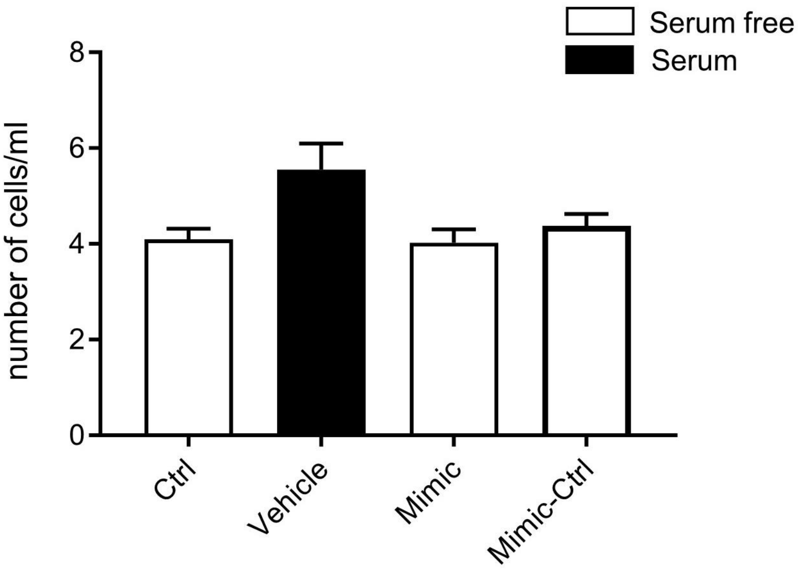

Supplementary Figure 4. Number of cells/ml 24 h after transfection. HT-29 cells were transfected with Mimic-Ctrl (50 nM) or miR-40-5p mimic (50 nM) in 24-wells plate. 24 h later cells were collected for counting and then used for migration and invasion assay.
